# Reevaluation of adverse drug reactions of psychiatric drugs under the chinese drug volume-based procurement policy

**DOI:** 10.1186/s12913-022-07851-4

**Published:** 2022-03-31

**Authors:** Zhiqiang Du, Ying Jiang, Yuan Shen, Qin Zhou, Shushan Wang, Haohao Zhu, Yingying Ji

**Affiliations:** 1 Wuxi Mental Health Center of Nanjing Medical University, Wuxi, Jiangsu, 214151 China; 2 Wuxi Tongren Rehabilitation Hospital, Wuxi, Jiangsu, 214151 China

**Keywords:** 4 + 7, Psychiatric drugs, Adverse drug reactions, Rational drug use

## Abstract

**Background:**

The "4 + 7" volume-based procurement is a "large group purchase" led by the Chinese government, with the aim of reducing the price of medicines by trading volume for price. Although the "4 + 7" drugs had passed the national consistency evaluation, the adverse drug reactions need to be further evaluated to ensure the safety of the "4 + 7" drugs with low prices. We aimed to analyze the occurrence characteristics and related influencing factors of adverse reactions of psychiatric drugs under the chinese drug volume-based procurement policy(4 + 7 policy), and provide references for clinical medication.

**Methods:**

137 cases of adverse drug reactions of four psychotropic drugs reported under the "4 + 7" policy in Wuxi Mental Health Center in 2020 were collected. The gender and age of patients, related "4 + 7" drugs, involving organs / systems, clinical manifestations, distribution of new / serious adverse reactions, clinic outcomes were analyzed.

**Results:**

Among the 137 cases of adverse drug reactions, the incidence of adverse drug reactions was the highest in patients aged 61–70 (25.38%). Mainly involved 4 "4 + 7" psychiatric drugs, of which olanzapine tablets caused the most adverse reactions (54, 39.24%). The adverse reactions mainly involved the digestive system, nervous system, cardiovascular system, blood and lymphatic system, among which the digestive system was the most common (61, 44.53%). A total of 8 cases (6.16%) of new and 26 cases of serious adverse reactions were reported, all of which led to the prolongation of disease course. Except for the transient side effects, most of that were improved or cured with no death, disability or teratogenicity after stopping or reducing the dose with symptomatic treatment.

**Conclusion:**

Since more and more drugs will be included in "4 + 7" for clinic, clinical pharmacists should strengthen the publicity and training of the knowledge of "4 + 7" drugs, strengthen the monitoring of adverse drug reactions, and provide timely feedback to the clinic, in order to achieve early prevention, early identification, timely diagnosis and reasonable intervention of the adverse drug reactions under the context of "4 + 7" policy.

## Introduction

In recent years, the cost of drugs in China has increased year by year, and drug expenditure has accounted for a large proportion in the medical expenditure of the total national and individual. As a big country of generic drugs, the development and use of high-quality and low-cost generic drugs has become the key to slowing down the excessive growth of drug costs. In order to solve the problems of inflated drug prices, decoupling of volume and price, lack of competition, scattered procurement, and lack of coordination in policies, in January 2019, the China issued the Drug Volume-based Procurement Policy, which was also called "4 + 7" policy (including 4 municipalities and 7 sub-provincial cities). A total of 25 basic drugs targeting different diseases were selected for the first time with the aim to reduce the price and improve the quality of medicines, transform and upgrade the pharmaceutical industry, deepen the reform of public hospitals and reduce the burden of medical security [1]. Of which, four psychiatric drugs were included in the first round of “4 + 7” policy and introduced to Wuxi at the end of 2019. Although the drugs that enter the "4 + 7" policy had passed the national consistency evaluation, the adverse drug reactions need to be further evaluated to ensure the safety of the "4 + 7" drugs with low prices [2–4].

In recent years, the issues of mental health have become an important public health issue over the world [5, 6]. The types and clinical use of psychotropic drugs increase continuously, as well as the duration of treatment. However, the widely pharmacological effects, complex interactions between psychiatric drugs and long-term medications lead to the increased adverse drug reactions [7, 8]. With the implementation of the national "4 + 7" policy, more and more domestic psychotropic drugs that have passed the national consistency evaluation have entered hospitals for clinic. With the widely application, new characteristics of adverse drug reactions of psychotropic drugs could be observed. However, no study related to the adverse drug reactions of “4 + 7” drugs has been reported. In this article, a retrospective analysis of the adverse reactions of “4 + 7” psychotropic drugs reported in our hospital in 2020 was conducted to discover the inherent characteristics and regularities of adverse reactions of such drugs, so as to provide evidence and reference for the safety and rational use of "4 + 7" psychotropic drugs.

## Materials and methods

A retrospective analysis was performed, and all 137 cases of adverse reactions related to four different "4 + 7" psychotropic drugs involved in 130 patients reported by the clinicians of Wuxi Mental Health Center to the National Adverse Reaction Monitoring Center from January 1, 2020 to December 31, 2020. The reports related to olanzapine, clozapine, risperidone and quetiapine were collected, and the reports related to other psychiatric drugs were removed. All collected reports were divided into two-level, including mild or moderate and severe adverse drug reactions. The mild/moderate means no obvious symptoms with no treatment required or significant symptoms and moderate damage to vital organs or systems, and the severe adverse drug reactions are defined as the vital organs or systems are serious damaged.

The patient’s gender, age, related "4 + 7" drugs, clinical manifestations, the involved organs / systems, distribution of new / serious adverse reactions and clinic outcomes were collected to examine the new features of adverse drug reactions of “4 + 7” psychotropic drugs. The institutional review board approval or patient informed consent was waived by the Ethics Committee of Wuxi Mental Health Centre. All methods were performed in accordance with relevant guidelines and regulations.

Descriptive statistics were used. Data was inputted to EpiData 3.1 software and statistical analyzed through SPSS 20.0. The counting data was described as the number of cases (composition ratio), and the measurement data was described by $\overline{x }$±s.

## Results

The distribution of gender and age of patients.

Among the 137 cases of adverse drug reactions, 130 patients were involved, of which 63 males, accounting for 48.46%; 67 females, accounting for 51.54%. The youngest patient was 14 years old, the oldest was 97 years old, with the average age of 59 years old. The patients aged from 61 to 70 had the highest incidence of adverse drug reactions, with the ratio of 25.38% (Table 1).

**Table 1:** Distribution of gender and age of the patients

Gender	Age									
	≤ 17	18–30	31–40	41–50	51–60	61–70	71–80	≥ 81	Total	Ratio (%)
Male	2	5	2	8	10	19	11	6	63	48.46
Female	2	7	3	10	10	14	9	12	67	51.54
Total	4	12	5	18	20	33	20	18	130	-
Ratio (%)	3.08	9.23	3.85	13.85	15.38	25.38	15.38	13.85	-	100.00

Details of the adverse drug reactions and related drugs.

A total of 4 "4 + 7" psychotropic drugs were involved in the adverse reaction reports of this study. Among them, olanzapine caused the most adverse reactions, 54 cases (39.24%), followed by clozapine in 38 cases (27.73%), risperidone in 23 cases (16.79%), and quetiapine in 22 cases (16.06%). Some drugs may involve multiple clinical manifestations of adverse reactions, so the number of specific adverse reactions was greater than the total number of reported cases (Table 2).

**Table 2:** "4 + 7" drugs causing adverse reactions and their clinical manifestations

Drugs	Case	Ratio (%)	Clinical manifestations (Case)
Olanzapine	54	39.42	Constipation (18) / Excessive sedation (6) / Extrapyramidal reactions (4) / Lower extremity edema (4) / Abnormal liver function (3) / Decreased blood pressure (3) / Dyslipidemia (2) / Sinus bradycardia (2) / Seizures (1) / Blurred vision (1) / Dry eyes (1) / Restlessness (1) / Prolonged QT interval (1) / Dry mouth (1) / Salivation (1) / Menstruation occurs two years after menopause (1) / Albumin reduction (1) / Hyperglycemia (1) / Allergic dermatitis (1) / Skin rash (1)
Clozapine	38	27.73	Constipation (17) / Salivation (6) / Sinus tachycardia (3) / Abnormal liver function (2) / Palpitation (1) / Epileptic seizures (1) / Akathisia (1) / Lethargy (1) / Incomplete intestinal obstruction (1) / decreased thyroid function (1) / Leukopenia (1) / Thrombocytopenia (1) / Increased blood concentration (1) / Frequent urination (1)
Risperidone	23	16.79	Extrapyramidal reactions (4) / Hyperprolactinemia (3) / Dizziness (2) / Leukopenia (2) / Akathisia (1) / Blurred vision (1) / drowsiness (1) / Excessive sedation (1) / Sinus bradycardia (1) / Sinus tachycardia (1) / Constipation (1) / Abnormal liver function (1) / Salivation (1) / Thrombocytopenia (1) / Urination difficulty (1) / Malignant syndrome (1)
Quetiapine	22	16.06	Constipation (4) / Leukopenia (3) / Abnormal liver function (3) / Excessive sedation (3) / Drowsiness (2) / Dizziness (1) / Dysphagia (1) / Dry mouth (1) / Hyperprolactinemia (1) / Lactation (1) / Asthenia (1) / Lower limb edema (1)
Total	137	100	

Note: Some drugs may involve multiple clinical manifestations of adverse reactions, so the number of specific adverse reactions was greater than the total number of reported cases

The organs/systems involved in adverse drug reactions and the clinical manifestations.

The adverse reactions reported in this study mainly involved the digestive system, nervous system, cardiovascular system, blood and lymph system, etc. The number of adverse reactions in the digestive system was 61 cases (44.53%), of which the number of constipation reports was 40 cases (29.20%). Some drugs may affect multiple organs / systems, so the number of specific adverse reactions ass greater than the total number of reported cases (Table 3).

**Table 3:** The organs/systems involved in adverse drug reactions and the clinical manifestations

Involved organs/systems	Case	Ratio (%)	Clinical manifestations (Case)
Digestive system	61	44.53	Constipation (40) / Abnormal liver function (9) / Salivation (8) / Dry mouth (2) / Incomplete intestinal obstruction (1) / Dysphagia (1)
Nervous system	33	24.09	Excessive sedation (10) / Extrapyramidal reactions (8) / Drowsiness (4) / Dizziness (3) / Epileptic seizures (2) / Akathisia (2) / Blurred vision (2) / Dry eyes (1) / Restlessness (1)
Cardiovascular system	12	8.75	Sinus tachycardia (4) / Sinus bradycardia (3) / Lower blood pressure (3) / Prolonged Q-T interval (1) / Palpitation (1)
Blood and lymphatic system	10	7.30	Leukopenia (6) / Thrombocytopenia (2) / Decreased albumin (1) / Increased blood drug concentration (1)
Endocrine system	7	5.11	Hyperprolactinemia (4) / Hypothyroidism (1) / Menstruation occurs two years after menopause (1) / Lactation (1)
Systemic reaction	7	5.11	Lower limb edema (5) / Malignant syndrome (1) / Asthenia (1)
Metabolic and nutritional disorders	3	2.19	Dyslipidemia (2) / Hyperglycemia (1)
Urinary and reproductive systems	2	1.46	Difficulty urinating (1) / Frequent urination (1)
Skin and accessory organs	2	1.46	Allergic dermatitis (1) / Rash (1)
Total	137	100	

Note: Due to some adverse reactions involved multiple organs / systems, the number of specific adverse reactions was more than the number of reported adverse reactions

Distribution and clinical manifestations of new / serious adverse reactions.

Among the 137 cases of adverse reactions of "4 + 7" psychotropic drugs reported in this study (Table 4), 8 cases were new (6.16%) and 26 were severe (20.00%) adverse reactions. Of these, olanzapine was found with the most new / serious adverse drug reactions of 12 cases (9.22%), followed by risperidone and clozapine of 8 cases (6.16%), and quetiapine was at least, with 6 cases (4.62%).

**Table 4:** Distribution and clinical manifestations of new / serious adverse drug reactions

Drugs	New			Severe		
	Case	Ratio (%)	Clinical manifestations (Case)	Cases	Ratio (%)	Clinical manifestations (Case)
Olanzapine	3	2.31	Allergic dermatitis (1) / Menstruation occurs two years after menopause (1) / Blurred vision (1)	9	6.91	Abnormal liver function (2) / Excessive sedation (1) / Rash (1) / Orthostatic hypotension (1) / Bradycardia (1) / Allergic dermatitis (1) / Dyslipidemia (1) / Extrapyramidal reaction (1)
Risperidone	3	2.31	Dizziness (2) / Sinus tachycardia (1)	5	3.85	Leukopenia (1) / Sinus bradycardia (1) / Malignant syndrome (1) / Blurred vision (1) / Difficulty urinating (1)
Clozapine	2	1.54	Hypothyroidism (1) / Increased blood drug concentration (1)	6	4.62	Incomplete intestinal obstruction (1) / Epileptic seizures (1) / Sinus tachycardia (1) / Abnormal liver function (1) / Thrombocytopenia (1) / Increased blood drug concentration (1)
Quetiapine	0	0		6	4.62	Abnormal liver function (2) / Leukopenia (1) / Excessive sedation (1) / Dry mouth (1) / Lactation (1)
Total	8	6.16		26	20.00	

The outcomes of adverse drug reactions.

Except for the transient adverse reactions in 137 cases, most of the adverse reactions were improved or cured by stopping or reducing the dose with symptomatic treatment. 115 cases (88.46%) improved, 13 cases (10.00%) did not improve, 2 cases (1.54%) recovered with no death, disability or teratogenicity (Table 5).

**Table 5:** The outcomes of adverse drug reactions (case/%)

Drugs	Recovered	Improved	Not improved	Total
olanzapine	1 (0.77)	45 (34.62)	5 (3.85)	51 (39.24)
clozapine	0 (0)	33 (25.38)	4 (3.08)	37 (28.46)
risperidone	1 (0.77)	17 (13.08)	3 (2.30)	21 (16.15)
Quetiapine	0 (0)	20 (15.38)	1 (0.77)	21 (16.15)
Total	2 (1.54)	115 (88.46)	13 (10.00)	130 (100)

## Discussion

Mental health is a major public health and social problem affecting economic and social development. Currently, patients with mental disorders are mostly treated with long-term medication, including antipsychotics, mood stabilizers, antidepressants and anxiolytics, etc. However, the adverse drug reactions related to those drugs directly affect the compliance and safety. Therefore, the adverse drug reactions of “4 + 7” psychiatric drugs need to be reevaluated to ensure the efficacy of such treatment.

In this study, the incidences of adverse drug reactions in females were slightly higher than that in males, which was consistent with the literatures related to the adverse reactions of psychotropic drugs before conducting the "4 + 7" policy [9]. Besides, in consistent with previously reports [10, 11], middle-aged and elderly people had a longer course of disease development and more complicated diseases than others, and different responsiveness and individual differences might be responsible for that related to the "4 + 7" psychotropic drugs.

The adverse drug reactions were mainly found among 4 psychotropic drugs in this study, of which olanzapine caused the most adverse reactions, followed by clozapine, risperidone and quetiapine. The results were consistent with that of a prospective study, which reported that the occurrence of adverse drug reactions was mainly existed in olanzapine and risperidone [12]. Besides, the adverse reactions of psychotropic drugs under "4 + 7" policy involved a wide range of organs/systems, the major organs / systems involved in were similar with that before "4 + 7" policy,. The digestive system (constipation) was the mainly involved by the adverse drug reactions of the psychotropic drugs under “4 + 7” policy, however, the nervous system (extrapyramidal adverse reactions) was the mainly involved before the “4 + 7” policy conducted [13–17], which may be caused by various factors, such as the production process of "4 + 7" drugs and the medication habits of clinicians. It also prompts that psychiatrist should pay attention to slowly increasing the dose or closely monitoring the blood drug concentration to keep the drug at the effective dose, as well as observe the sensitivity of patient against the drug and reduce the occurrence of adverse reactions.

New / serious adverse drug reactions are the most important information that affects the safety of medications [18]. Among the 137 cases of adverse drug reactions of "4 + 7" psychotropic drugs in this study, 6.16% of new adverse reactions and 20.00% of severe adverse reactions were reported. Among the new adverse reactions, 2 cases were severe with allergic dermatitis (caused by olanzapine), and significant increased blood drug concentration (caused by clozapine). Considering the complex mechanism of action of psychotropic drugs, the adverse reactions caused are varied and difficult to be predicted, therefore, the clinic should pay more attention to the new / serious adverse drug reactions of “4 + 7” drugs. In addition to transient adverse reactions in the 137 cases of adverse reactions, the vast majority of adverse reactions improved or healed by stopping or reducing the dose combined with symptomatic treatment, which further proved that strictly monitor the adverse drug reactions and medication could improve the clinical application of “4 + 7” drugs [19].

Although presenting the importance of reevaluation the adverse drug reactions of “4 + 7” drugs, our study had some limitations. First, the small sample size, which may lead to the bias of the results. Second, since the study was a retrospective analysis and the adverse drug reactions were filled in and reported by clinicians spontaneously, there may be omissions or underreporting. Future investigation will expand the sample size to verify the result of the study, and establish incentives to encourage the clinicians pay more attention to the adverse drug reactions of “4 + 7” drugs.

## Conclusion

To sum up, the unclear mechanism of mental illness may lead to the complex adverse drug reactions of psychotropic drugs during the course of treatment [20–22]. This study retrospective analyzed the adverse drug reactions under the “4 + 7” policy, 4 psychotropic drugs (olanzapine, clozapine, quetiapine, and risperidone) were mainly involved. The results showed that adverse reactions were related to the gender and age of patients, among which the middle-aged and elderly female had higher incidence. The adverse drug reactions could affect almost all organs, mainly involved the digestive system, nervous system, cardiovascular system, blood and lymphatic system.

As more and more "4 + 7" drugs entering the clinical use, although the effectiveness has been confirmed through the national consistency evaluation, their adverse drug reactions and safety are urgently needed to be further verified. To achieve the aim of “4 + 7” policy, the clinical pharmacists should strengthen the propaganda and training of "4 + 7" drug knowledge, strengthen the monitoring of adverse drug reactions and timely feedback to the clinic, so as to achieve early prevention, early identification, timely diagnosis and reasonable intervention of adverse drug reactions to ensure the safety of “4 + 7” drugs. Besides, during the medication, the history of adverse drug reactions of patients should be evaluated, and special populations and psychiatric patients with multiple physical diseases should be closely monitored for the adjustment of the dose and combination medication to avoid or reduce adverse reactions.

### Ethics approval and consent to participate

This study did not require Institutional Review Board approval or patient informed consent because the monitoring of adverse drug reactions had been a routine work of the hospital according to the Chinese policy, which was waived by the Ethics Committee of Wuxi Mental Health Centre. All methods were performed in accordance with relevant guidelines and regulations.

## Declarations

### Availability of data and materials

The dataset generated during and analysed during the current study are available from the corresponding author on reasonable request.
